# Quantifying the Thresholds of the Phospholipid Surface Density for Nonspecific Protein Adsorption and Desorption at the Triacylglycerol/Water Interface

**DOI:** 10.1021/acs.langmuir.5c03216

**Published:** 2025-09-12

**Authors:** Chiho Kataoka-Hamai

**Affiliations:** Research Center for Macromolecules and Biomaterials, 52747 National Institute for Materials Science, Tsukuba, Ibaraki 305-0044, Japan

## Abstract

Quantitative investigation of protein adsorption and desorption at triacylglycerol/water interfaces is of great importance for understanding the properties and functions of intracellular lipid droplets. In this study, we investigated the adsorption and desorption of cytochrome c, lysozyme, and bovine serum albumin at the tricaprylin/water interface covered with 1,2-dioleoyl-*sn-glycero*-3-phosphocholine (DOPC) using a surface pressure measurement system and a pendant drop tensiometer. The aim of this study was to understand the quantitative relationships between the area per DOPC molecule and the protein adsorption and desorption. We found that the area per DOPC molecule required to preclude bovine serum albumin adsorption was much smaller than that required to preclude the adsorption of cytochrome *c* or lysozyme. However, the area per DOPC molecule required for all of the bound protein molecules to desorb from the interface was independent of the type of protein, and this threshold area per DOPC molecule was in good agreement with an area per lipid value previously reported for fully hydrated DOPC bilayers.

## Graphical abstract

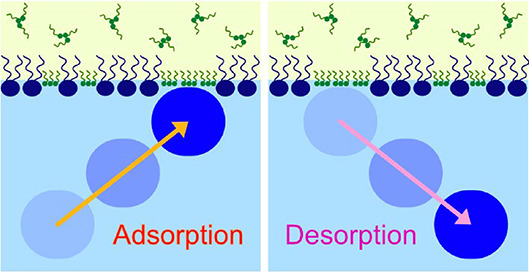

## Introduction

Lipid droplets (LDs) are intracellular organelles that store neutral lipids, mainly triacylglycerols (TAGs) and cholesteryl esters, within their core. This core is surrounded by a phospholipid monolayer decorated with various proteins.^1^ Previous studies have identified 100–150 distinct proteins located on the LD surface.^2–4^ These proteins attach to the LD surface through at least two pathways. Depending on the pathway, the proteins are grouped into class I and class II proteins.^1^ Class I proteins migrate from the endoplasmic reticulum membrane to the LDs through endoplasmic reticulum–LD bridges.^5,6^ In contrast, class II proteins, which generally contain amphipathic helices, migrate to the LDs from the cytosol. Computational studies have shown that the amphipathic helices recognize and preferentially associate with the phospholipid packing defects where neutral lipids are exposed to the cytosol.^7,8^

The size of LDs dynamically changes through lipolysis and lipogenesis in response to the metabolic conditions.^9^ The size change is thought to regulate the protein composition on the LD surface. For instance, when a LD shrinks, weekly associated proteins may dissociate from the LD owing to a decrease in the number of packing defects and enhanced molecular crowding.^1^ Considering this regulation mechanism of the protein composition, as well as the binding mechanism of class II proteins, it is clear that phospholipid packing defects play a crucial role in the LD functions. The surface area of defects varies with the phospholipid surface density.^10^ Thus, in the present study, we focused on the effect of the phospholipid surface density on protein adsorption and desorption.

A recent study investigated the influence of the phospholipid surface density on the binding of perilipins, the most abundant LD proteins.^11^ The results indicated that the binding of different perilipins to LDs is differently affected by the phospholipid surface density. However, the quantitative correlation of protein binding with the phospholipid surface density remains poorly understood. In this study, therefore, we investigated the relationship between the phospholipid surface density at a TAG/water interface and protein adsorption and desorption using a quantitative approach.

The phospholipid monolayer surrounding the LD core acts as a barrier to non-LD-binding proteins. However, the minimum phospholipid surface density required to prevent the adsorption of nonspecific proteins is not well understood. Thus, in this study, we investigated the nonspecific adsorption and desorption of three globular proteins, lysozyme, cytochrome c, and bovine serum albumin (BSA), at a TAG/water interface. The main phospholipids on LDs are phosphatidylcholines (PCs).^12,13^ Therefore, we studied the interface covered with a PC monolayer.

We determined two thresholds of the PC surface density. At low PC surface densities (Figure 1A­(i)), many packing defects are available for protein adsorption. As the PC surface density increases, the number of these defects progressively decreases and protein adsorption is eliminated (Figure 1A­(ii)). We define this critical area per PC molecule as *A*_a_. We also determined another threshold by considering the case in which protein molecules are already bound to the interface (Figure 1B­(i)). In this case, as the PC surface density increases, protein molecules begin to dissociate from the interface, and, eventually, all of the protein molecules are displaced into the aqueous phase (Figure 1B­(ii)). We define this critical area per PC molecule as *A*_d_. *A*_a_ and *A*_d_ can be different values because they are determined for different protein conformations. *A*_a_ is determined for undenatured protein in the aqueous phase whereas *A*_d_ is determined for denatured protein at the interface.

**Figure 1:**
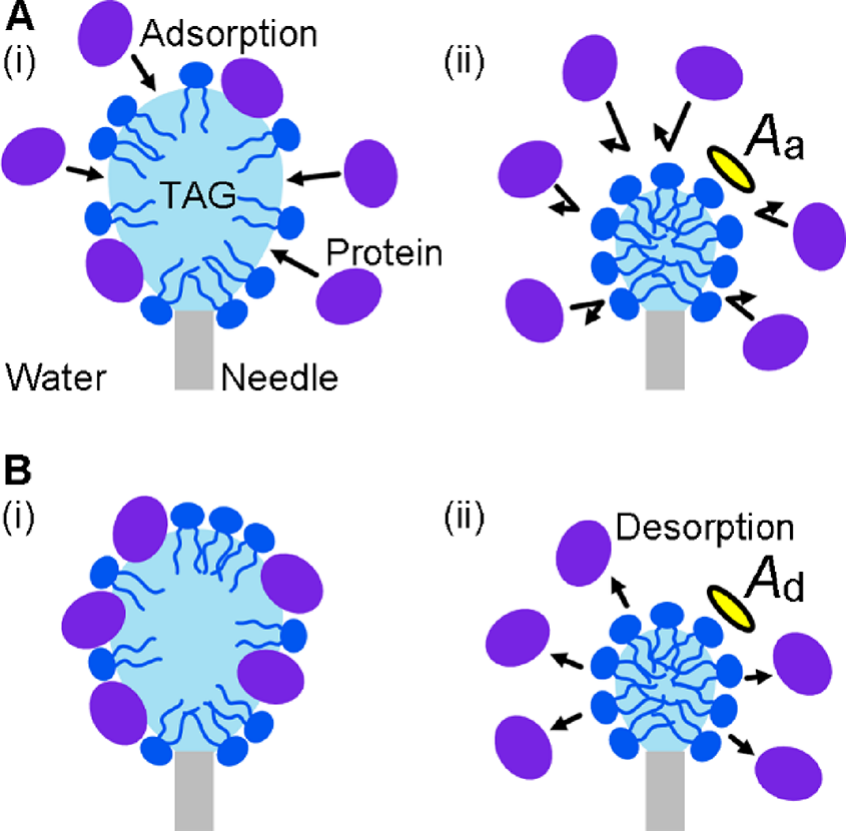
Measurement of protein adsorption and desorption at a triacylglycerol (TAG)/water interface using pendant drop tensiometry. A TAG drop is pinned at the end of a needle immersed in the aqueous phase. For detecting (A) the inhibition of protein adsorption and (B) the complete desorption of bound protein molecules, the area per phospholipid molecule is decreased by decreasing the drop volume. (A) Measurement of the threshold area per phospholipid molecule that prevents protein adsorption (*A*_a_). (i) An interface with low phospholipid surface density allows protein adsorption. (ii) Once the area per phospholipid molecule reaches *A*_a_, protein molecules cannot bind. (B) Measurement of the threshold area per phospholipid molecule at which adsorbed protein molecules are completely displaced into the aqueous phase (*A*_d_). (i) The interface is initially covered with phospholipid and protein molecules. (ii) Once the area per phospholipid molecule reaches *A*_d_, all of the protein molecules are eliminated.

We demonstrate a method to determine *A*_a_ and *A*_d_ using pendant drop tensiometry. Using this method, we studied the adsorption and desorption of lysozyme, cytochrome *c*, and BSA at a tricaprylin/water interface covered with 1,2-dioleoyl-*sn-glycero*-3-phosphocholine (DOPC). The results showed that the *A*_a_ value for BSA was much smaller than those for lysozyme and cytochrome c, whereas the *A*_d_ values for the three proteins were almost the same.

## Experimental Section

### Materials

DOPC (>99%) was purchased from Avanti Polar Lipids (Alabaster, AL, USA). The DOPC stock solutions were prepared in chloroform, and their concentrations were determined by phosphorus assay.^14^ Tricaprylin (≥99%) and cytochrome c from equine heart (≥95%) were purchased from Sigma-Aldrich (St. Louis, MO, USA). BSA (≥98%) and lysozyme from egg white were purchased from FUJIFILM Wako Pure Chemical Corporation (Osaka, Japan). All of the chemicals were used as received. Phosphate buffered saline (PBS) buffer (137 mM NaCl, 2.7 mM KCl, 10 mM Na_2_HPO_4_, 2 mM KH_2_PO_4_, pH 7.4) was used for all of the experiments.

### Vesicle Preparation

The vesicles were prepared from a DOPC stock solution. Bulk chloroform was removed under a nitrogen stream. Any remaining solvent was evaporated under vacuum. PBS buffer was added to give a DOPC concentration of 5 mM. The samples were subjected to 10 freeze–thaw cycles (liquid nitrogen/room temperature) and subsequently extruded 11 times through a polycarbonate membrane filter (100 nm pores) using a mini-extruder (Avanti Polar Lipids).

### Surface Pressure Measurements

The surface pressure at the air/buffer interface was measured using a platinum Wilhelmy plate (perimeter 20 mm) with a KSV NIMA system (Biolin Scientific, Gothenburg, Sweden) equipped with a Langmuir trough (35 cm × 7.5 cm) and two barriers. Lipid in chloroform (∼1 mM) was spread onto the buffer surface (23 °C). The buffer temperature was kept constant by using a water circulator. After solvent evaporation for 2 min, DOPC monolayers containing 0–40% tricaprylin and tricaprylin monolayers were compressed at a rate of 15 mm/min. The compression rates for the DOPC monolayers containing 50 and 60% tricaprylin were 45 and 75 mm/min, respectively, because these monolayers were unstable under slow compression (Figure S1). The DOPC/tricaprylin ratios are expressed as molar percentages.

### Pendant Drop Tensiometry

The interfacial tension at the tricaprylin/buffer interface was measured with a DSA25 drop shape analyzer (Krüss GmbH, Hamburg, Germany). A tricaprylin drop was formed at the end of a J-shaped needle immersed in buffer in a quartz cell (base size 10 mm × 20 mm, height 45 mm). Images of the drop were recorded under light-emitting diode lamp illumination. The drop shapes were fit to the Young–Laplace equation using ADVANCE software (Krüss GmbH). For the *A*_a_ and *A*_d_ measurements, the unbound lipids and proteins were removed by flowing buffer through the quartz cell at a rate of 2 mL/min using a peristaltic pump and a suction pump (Figure S2). During this washing process, the continuous phase was stirred using a small magnetic stirrer (length 5 mm). When protein was added to the cell for protein adsorption, the solution was stirred with a magnetic stirrer to give a homogeneous protein concentration (Figure S2).

### Experimental Values

The reported values are the mean (±standard error) of *N* determinations. The definitions of the symbols used for the measured values (*A*_a_, *A*_d_, Π, Π_0_, γ, γ_0_, Δγ_pads_, γ_i_, Δγ, γ_i_′, Δγ′, Δγ_b_, γ_i,0_, and γ_bp_) are summarized in Table S1.

## Results and Discussion

### Dependence of the Interfacial Tension on the Area per DOPC Molecule at the Tricaprylin/Buffer Interface

To determine the threshold areas per DOPC molecule (*A*_a_ and *A*_d_) from the interfacial tension data, we first determined the relationship between the area per DOPC molecule and the interfacial tension (Figure 2) using our previously reported method.^15,16^ First, we measured the surface pressures of DOPC/tricaprylin monolayers at the air/buffer interface using a Langmuir trough (Figure 2A). When the DOPC concentration was 40% (purple), the surface pressure smoothly increased from nearly zero upon compression. In this region, all of the tricaprylin molecules were within the DOPC monolayer. However, when the surface pressure reached ∼22 mN/m, the slope of the curve abruptly changed. At this shoulder, tricaprylin molecules seemed to start to move out of the monolayer to form a new bulk phase.^16,17^ The surface pressure data after the shoulder overlapped with other data obtained at different DOPC concentrations. This is because the tricaprylin concentration in the DOPC monolayer was determined by the area per DOPC molecule independently of the DOPC/tricaprylin ratio in the lipid mixture initially spread on the buffer surface.^16,17^ This one-to-one correspondence between the tricaprylin concentration in the monolayer and the area per DOPC molecule resulted from the removal of excess tricaprylin molecules from the monolayer to the bulk phase. The overlapped surface pressure data (Π) were used to correlate the interfacial tension at the tricaprylin/buffer interface (γ) with the area per DOPC molecule using the following equation:^16^

$$\gamma =\gamma_{0}+\Pi_{0}-\Pi$$ 1

where γ_0_ is the interfacial tension at the pure tricaprylin/buffer interface and Π_0_ is the collapse pressure of the pure tricaprylin monolayer at the air/buffer interface. γ_0_ was determined to be 24.3 (±0.1) mN/m (*N* = 7) by pendant drop tensiometry. Π_0_ was determined to be 20.0 (±0.1) mN/m (*N* = 4) using the Langmuir trough (Figure 2B). Using eq 1, we converted the surface pressure data for the air/buffer interface (Figure 2A) to the interfacial tension data for the tricaprylin/buffer interface (Figure 2C).

**Figure 2:**
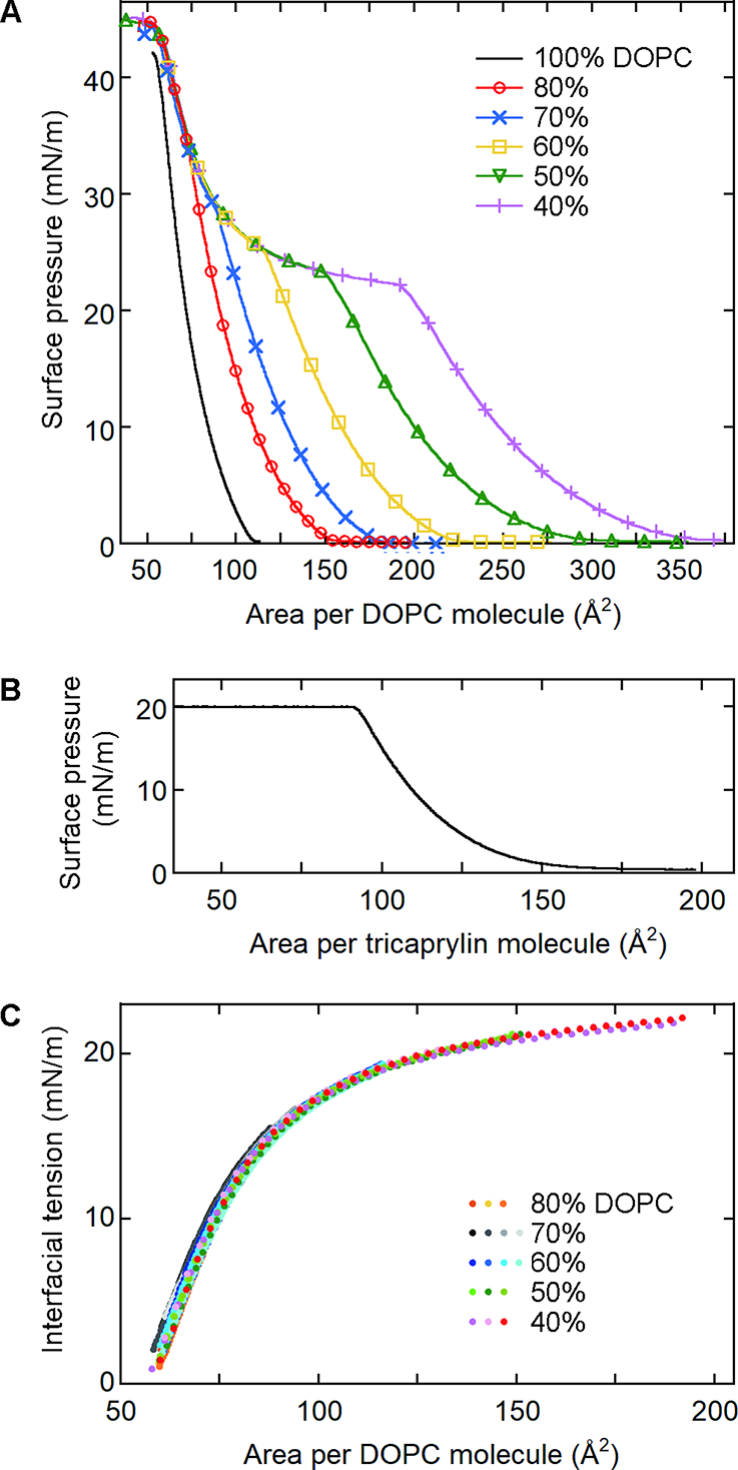
Determination of the dependence of the interfacial tension on the area per 1,2-dioleoyl-*sn-glycero*-3-phosphocholine (DOPC) molecule at the tricaprylin/buffer interface. (A) Surface pressure data for the air/buffer interfaces covered with DOPC monolayers containing 0–60% tricaprylin. (B) Surface pressure data for tricaprylin at the air/buffer interface. (C) Dependence of the interfacial tension at the tricaprylin/buffer interface on the area per DOPC molecule. For each DOPC concentration, we obtained three to four sets of data (different colors).

### Protein Adsorption at the Pure Tricaprylin/Buffer Interface

We investigated the adsorption of the proteins (2 μM) at the clean tricaprylin/buffer interface using pendant drop tensiometry (Figure 3) by measuring the interfacial tension difference between the clean interface and the interface incubated with the protein for 30 min (Δγ_pads_). BSA showed the largest Δγ_pads_ value, whereas lysozyme and cytochrome c showed similar but lower Δγ_pads_ values (Table 1). These results indicate that BSA most strongly bound to the interface and lysozyme and cytochrome c more weakly bound to the interface with a similar strength.

**Figure 3:**
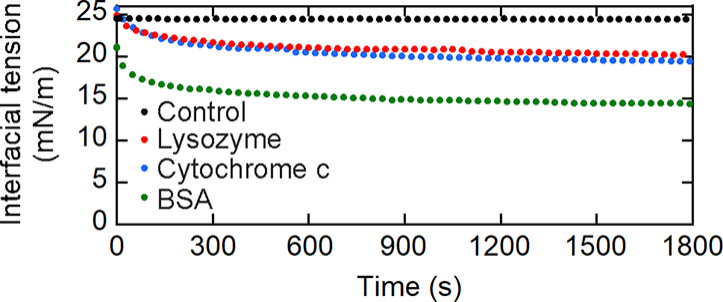
Interfacial tension data for tricaprylin drops exposed to pure buffer (black), 2 μM lysozyme (red), 2 μM cytochrome *c* (blue), and 2 μM bovine serum albumin (BSA) (green).

**Table 1:** Δγ_pads_, *A*_a_, and *A*_d_ for Lysozyme, Cytochrome *c*, and BSA Adsorption at the Tricaprylin/Buffer Interface (*N* = 3–8)

protein	Δγ_pads_ (mN/m)	*A*_a_ (Å^2^)	*A*_d_ (Å^2^)
lysozyme	–4.7 (±0.3)	88.3 (±1.4)	66.9 (±0.3)
cytochrome *c*	–4.6 (±0.1)	87.4 (±0.04)	66.7 (±0.6)
BSA	–10.1 (±0.1)	69.8 (±0.3)	68.1 (±0.5)

### Determination of the Threshold Area Per DOPC Molecule (*A*_a_)

We determined the *A*_a_ values at which protein adsorption was completely prevented (Figure 1A). We first prepared DOPC-bound interfaces with different DOPC surface densities and then added the protein to measure the interfacial tension change due to the protein adsorption. These measurements were performed by two methods. In the first method (Figure 4A), a tricaprylin drop was exposed to a vesicle solution (0.07 mM lipid). After the DOPC monolayer formed, the excess vesicles were removed by flowing buffer into the measurement cell for 10 min with gentle stirring using a magnetic stirrer (Figure 4A, wash). After the washing step, the buffer flow and stirring were stopped to measure the interfacial tension under the static condition (γ_i_, Figure 4A). Different γ_i_ values were obtained by changing the vesicle adsorption time. We subsequently added protein to give a concentration of 2 μM with gentle stirring for the first 3 min (Figure 4A, protein adsorption). The protein adsorption was monitored for 30 min. We then calculated the interfacial tension difference between the γ_i_ value and the value recorded 30 min after the addition of the protein (Δγ).

**Figure 4:**
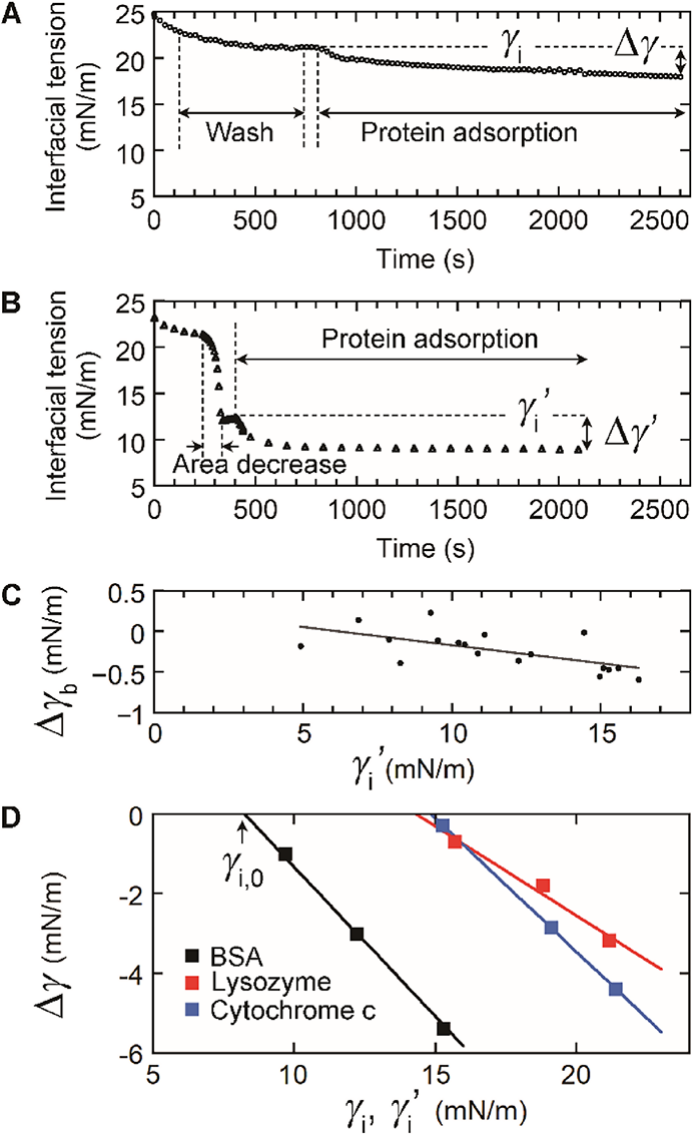
Determination of the threshold area per DOPC molecule (*A*_a_). Two methods were used to measure the interfacial tension change (Δγ) caused by the adsorption of the protein (2 μM) at the DOPC-covered tricaprylin/buffer interface for 30 min. (A) Method used for obtaining an interfacial tension of >16 mN/m before protein adsorption. The data for lysozyme are shown. After the adsorption of DOPC vesicles (0.07 mM lipid), the excess lipids were removed to measure γ_i_ (wash), and the protein was adsorbed for 30 min (protein adsorption). The interfacial tension decrease after the addition of the protein is denoted as Δγ. (B, C) Method used for obtaining an interfacial tension of ≤16 mN/m before protein adsorption. The data for BSA are shown. (B) After DOPC adsorption (0.07 mM lipid), the surface area of the tricaprylin drop was decreased to obtain the γ_i_′ value (area decrease). Without removing the unbound vesicles, the protein (2 μM) was adsorbed for 30 min (protein adsorption). The interfacial tension decrease after the addition of the protein is denoted as Δγ′. (C) Interfacial tension change (Δγ_b_) in the absence of the protein after decreasing the drop volume in method B. The data (dots) are fitted to a linear equation (line). The Δγ_b_ value at a given γ_i_′ value was estimated from this linear relationship. Δγ was obtained by subtracting Δγ_b_ from Δγ′ (eq 2). (D) Determination of the interfacial tension before protein addition that gives Δγ = 0 (γ_i,0_). The arrow indicates γ_i,0_ for the BSA data. The area per DOPC molecule at an interfacial tension of γ_i,0_ is *A*_a_.

The above method was used for γ_i_ > 16 mN/m (Figure 4A). For ≤16 mN/m, we used the following method (Figure 4B) because the vesicle adsorption was too slow to achieve γ_i_ ≤ 16 mN/m. After DOPC monolayer formation, the drop volume was decreased to reduce the interfacial tension to γ_i_′ (Figure 4B, area decrease). The values of γ_i_′ were recorded without removing free DOPC vesicles. The protein was then added to give a concentration of 2 μM. The protein adsorption was performed for 30 min with gentle stirring for the first 3 min (Figure 4B, protein adsorption). For the data analysis, we calculated the interfacial tension difference between the γ_i_′ value and the value recorded 30 min after the addition of the protein (Δγ′, Figure 4B). We did not remove the free vesicles during the measurements because the adsorption of DOPC at the interface with interfacial tension of ≤16 mN/m was relatively slow. To extract the interfacial tension change caused solely by protein adsorption (Δγ), we subtracted the interfacial tension change due to DOPC adsorption from Δγ′ as follows:

$$\Delta \gamma =\Delta \gamma^{\prime }-\Delta \gamma_{b}$$ 2

where Δγ_b_ is the interfacial tension change predicted to occur owing to the adsorption of DOPC. Δγ_b_ was measured without protein after the drop volume decrease. The Δγ_b_ – γ_i_′ relationship was fitted to a linear function (Figure 4C, line). This linear approximation was used to estimate the Δγ_b_ value at a given γ_i_′ value.

Using the results obtained using the above two methods (Figure 4A–C), we determined the Δγ values at different γ_i_ (or γ_i_′) values for the three proteins (Figure 4D, squares). The data were well fitted to linear relationships (Figure 4D, lines), which were used to calculate the interfacial tension values at Δγ = 0 (γ_i,0_, Figure 4D). γ_i,0_ is the interfacial tension at an area per DOPC molecule of *A*_a_ (Figure 1A). Therefore, we determined the *A*_a_ values from the γ_i,0_ data by using the interfacial tension dependence on the area per DOPC molecule in Figure 2C. The results showed that *A*_a_ was the smallest for BSA, and the *A*_a_ values for lysozyme and cytochrome *c* were larger but similar (Table 1). The results suggest that BSA is the most capable of adsorbing to tricaprylin exposed to the aqueous phase through DOPC packing defects, whereas lysozyme and cytochrome c are less capable of adsorbing to tricaprylin. These results were consistent with the Δγ_pads_ data (Table 1), which showed that BSA was the most strongly bound to the pure interface.

### Determination of the Threshold Area per DOPC Molecule (*A*_d_) for BSA

We investigated the threshold area per DOPC molecule at which the bound protein completely desorbed from the interface (*A*_d_, Figure 1B). We first studied BSA (Figure 5A). The experiments consisted of the following two processes: the formation of the interface that bound DOPC and BSA (Figure 1B­(i)) and decreasing the surface area to desorb BSA (Figure 1B­(ii)). In the first process (Figure 5A), vesicle adsorption (0.07 mM DOPC) was carried out, followed by decreasing the drop volume (Figure 5A, area decrease) to achieve a desired interfacial tension value (γ_bp_). This interface was incubated with BSA (2 μM) for 10 min (Figure 5A, BSA adsorption) with gentle stirring for the first 3 min. The number of adsorbed DOPC molecules on the interface was estimated from the γ_bp_ value using the interfacial tension data in Figure 2C. This estimated number of adsorbed DOPC molecules was assumed to be constant throughout the experiment. During the adsorption of BSA at the interface, the adsorption of DOPC was negligible because the γ_bp_ values were small (<10 mN/m). After the adsorption of BSA, the unbound BSA and vesicles were removed by flowing buffer through the measurement cell for 10 min with gentle stirring (Figure 5A, wash).

**Figure 5:**
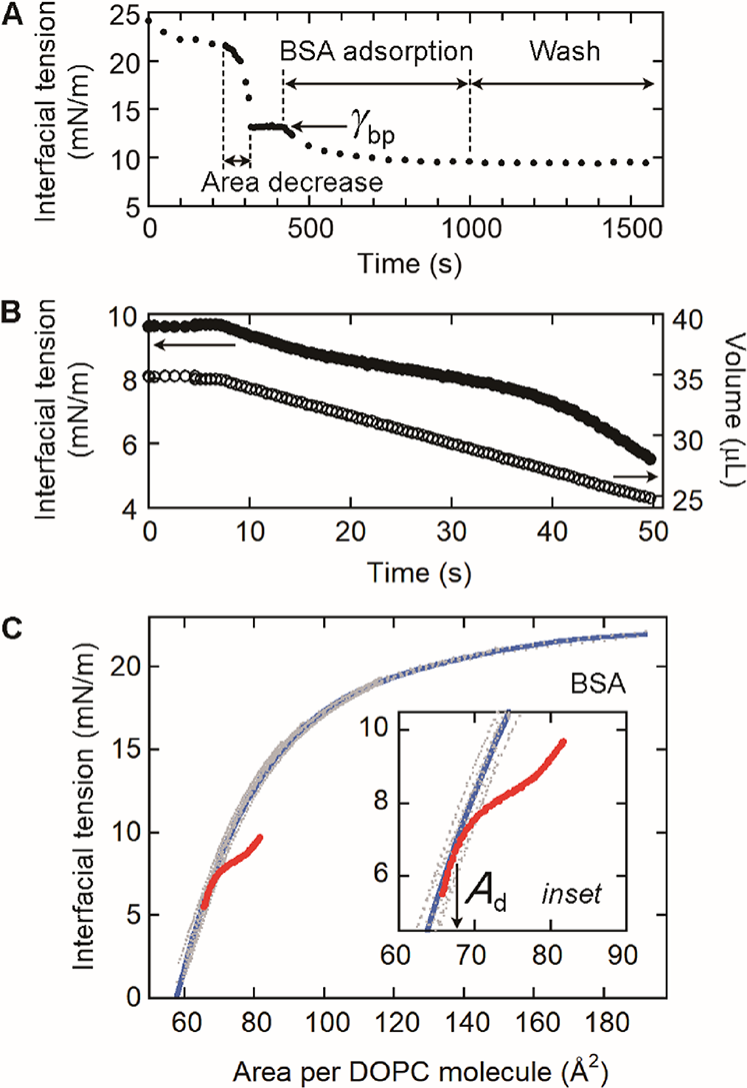
Determination of the threshold area per DOPC molecule (*A*_d_) for BSA. (A) Formation of a tricaprylin/buffer interface adsorbing DOPC and BSA. After DOPC adsorption (0.07 mM lipid), the interfacial tension was decreased to γ_bp_ by decreasing the volume of the tricaprylin drop (area decrease). The interface was incubated with BSA (2 μM) for 10 min (BSA adsorption), followed by washing with buffer for 10 min (wash). (B) Measurement of the interfacial tension (solid circles) with decreasing drop volume (open circles) for the sample in (A). The measurement was continued until the tricaprylin drop detached from the needle tip. (C) Dependence of the interfacial tension on the area per DOPC molecule (red) obtained from the data in (B). The data for the protein-free interface in Figure 2C (gray data points) and their fitting curve (blue) are also given. The *A*_d_ value is the area per DOPC molecule at which the red and blue curves start to overlap (insert).

For this interface, we measured the interfacial tension (Figure 5B, solid circles) while decreasing the drop volume (Figure 5B, open circles) at a rate of 0.25 or 0.5 μL/s. We simultaneously recorded the drop surface area, which was used to calculate the area per DOPC molecule. Specifically, we divided the drop surface area by the number of bound DOPC molecules estimated from the γ_bp_ value. The interfacial tension data (Figure 5B, solid circles) were then plotted against the area per DOPC molecule (Figure 5C, red). The results showed that for area per DOPC molecule of >∼68 Å^2^, the interfacial tension was smaller than that obtained for the protein-free interface (Figure 5C, gray data points, blue fitting curve) owing to adsorbed BSA. For area per DOPC molecule of ≤∼68 Å^2^, however, the two curves recorded with (red) and without protein (gray data points, blue fitting curve) overlapped. The data indicated that the bound protein molecules progressively desorbed with decreasing area per DOPC molecule, and the protein was finally completely removed from the interface at an area per DOPC molecule of ∼68 Å^2^. This value is *A*_d_ (Table 1).

### Dependence of the Interfacial Tension on the Area Per DOPC Molecule after BSA Desorption

The above data suggested that BSA completely desorbed from the interface by decreasing the drop surface area. We confirmed this notion by experiments consisting of the following three processes. In the first process, we formed the interface that adsorbed DOPC and BSA, as described for the data in Figure 5A. In the second process, the drop volume was decreased (Figure 6A, step 1, red) until complete BSA desorption appeared to occur (Figure 6B, red). The desorbed BSA molecules were removed by flowing buffer through the measurement cell for 5 min with gentle stirring. In the final process, the interface was subjected to a drop volume change at a rate of 1 μL/s (Figure 6A, steps 2–7). The interfacial tension data obtained during these steps (Figure 6B, black, pink, light blue, orange, purple, and green) were in good agreement with the curve obtained for the protein-free DOPC monolayer (Figure 6B, gray data points, blue fitting curve). These results verified the complete removal of BSA from the interface during step 1.

**Figure 6:**
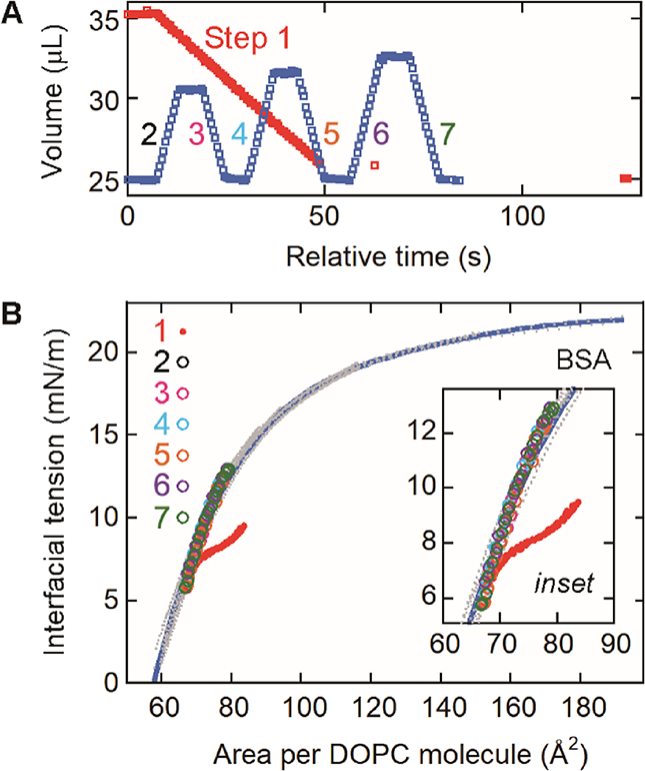
Interfacial tension data obtained for a tricaprylin/buffer interface that adsorbed DOPC and BSA. (A) Decreasing the drop volume to desorb BSA (red, step 1), followed by changing the volume to investigate the dependence of the interfacial tension on the area per DOPC molecule (blue, steps 2–7). The drop volume during these successive processes is plotted against the relative time. (B) Dependence of the interfacial tension on the area per DOPC molecule obtained during step 1 (red) and the subsequent steps 2–7 (black, pink, light blue, orange, purple, and green). The data for the protein-free interface in Figure 2C (gray data points) and their fitting curve (blue) are also shown.

### Determination of the Threshold Area per DOPC Molecule (*A*_d_) Values for Lysozyme and Cytochrome *C*

We next determined the *A*_d_ value for lysozyme. The experiments consisted of the following three processes: the formation of a lysozyme-bound DOPC monolayer, shrinking the drop to desorb lysozyme, and drop expansion and shrinkage cycles to measure the dependence of the interfacial tension on the area per DOPC molecule. The first process (monolayer formation) was performed by one of the following methods. To obtain γ_bp_ < 18 mN/m, we used the method used for BSA (Figure 5A). To obtain γ_bp_ > 18 mN/m, we used the following method (Figure 7A). After vesicle adsorption, the unbound lipids were removed by flowing buffer through the measurement cell for 10 min with stirring (Figure 7A, 1st wash). The interface was subsequently incubated with the protein (2 μM) for 10 min with stirring (Figure 7A, lysozyme adsorption), followed by the removal of the free protein molecules by flowing buffer through the measurement cell for 10 min with stirring (Figure 7A, 2nd wash).

**Figure 7:**
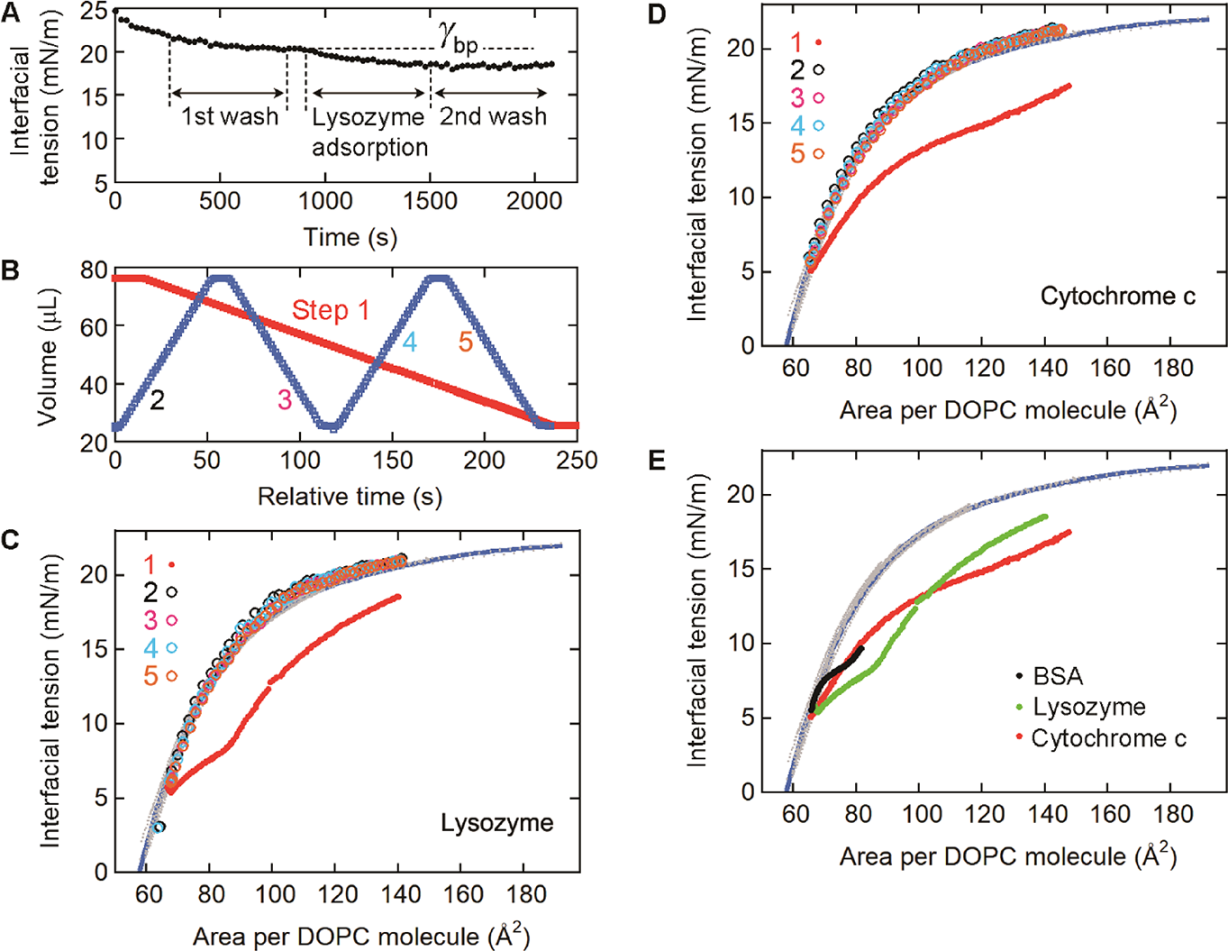
Determination of the threshold areas per DOPC molecule (*A*_d_) for (A–C) lysozyme and (D) cytochrome *c*, and (E) comparison of the results for the different proteins. (A) Formation of a tricaprylin/buffer interface covered with DOPC and lysozyme. The process consisted of DOPC adsorption (0.07 mM lipid), DOPC removal (first wash), lysozyme adsorption, and lysozyme removal (second wash). (B, C) Interfacial tension measurements for the interface in (A). First, the drop volume (B, red, step 1) was decreased to measure the interfacial tension change caused by the decrease in the area per DOPC molecule (C, red). Second, the drop volume was varied (B, blue, steps 2–5) to measure the interfacial tension change caused by cycles of an increase and a decrease in the area per DOPC molecule (C, black, pink, light blue, and orange). (D) Dependence of the interfacial tension on the area per DOPC molecule obtained for cytochrome c. Steps 1–5 (red, black, pink, light blue, and orange) were similar to those for the lysozyme data in B. (E) Comparison of the results for the different proteins. The BSA data in Figure 5C are overlaid with the lysozyme (C) and the cytochrome c (D) data. (C–E) Data in the absence of protein (Figure 2C, gray data points) and their fitting curve (Figure 2C, blue) are also shown.

The second process (drop shrinkage) and final process (drop shrinkage/expansion cycles) were performed in similar ways to those for BSA (Figures 5B,C and 6). For lysozyme desorption (Figure 7B, red, step 1), the volume of the drop to which DOPC and lysozyme adsorbed was decreased at a rate of 0.25 or 0.5 μL/s. The desorbed protein molecules were removed by flowing buffer for 5 min with gentle stirring. The interfacial tension during step 1 (Figure 7C, red) matched the data obtained without protein (Figure 7C, gray data points, blue fitting curve) at an area per DOPC molecule of ∼67 Å^2^. This area per DOPC molecule is considered to be the *A*_d_ value of lysozyme. After step 1, the drop volume was repeatedly increased and decreased at a rate of 1 μL/s (Figure 7B, blue, steps 2–5). The interfacial tension data during these steps (Figure 7C, black, pink, light blue, and orange) were in good agreement with those obtained for the pure DOPC monolayer (Figure 7C, gray data points, blue fitting curve). The results demonstrated that lysozyme was eliminated from the interface during step 1. We also determined the *A*_d_ value for cytochrome c in a similar manner (Figure 7D). The results showed that the *A*_d_ values for the three different proteins were similar despite the large differences in the *A*_a_ values (Figure 7E and Table 1).

### Discussion

The three proteins have different properties (Table 2), and they therefore have different interactions with tricaprylin and DOPC. We expect that these different interactions are the cause of the different Δγ_pads_ and *A*_a_ values (Table 1). However, detailed analyses are difficult because of the following reason. The total energy of the adsorption of proteins at oil/water interfaces is mainly affected by the contributions from electrostatic interactions, solvation forces, van der Waals interactions, and conformational changes.^18,19^ The first term (electrostatic interactions) needs to be considered because oil/water interfaces are generally negatively charged.^20–23^ The second term (solvation forces) is repulsive or attractive depending on the interacting surfaces.^19^ For example, when hydrophilic surfaces distributed on the protein interact with the hydrophilic DOPC headgroups, the protein experiences a repulsive force called the hydration force.^19^ However, when hydrophobic surfaces distributed locally on the protein interact with the hydrophobic tricaprylin surfaces exposed through DOPC packing defects, the protein experiences an attractive force.^19^ The final contribution (conformational changes) arises from the conformational rearrangement within the protein at the interface. After the conformational changes, the other contributions from the electrostatic interactions, solvation forces, and van der Waals interactions also change. Because of this complexity, it is not possible to infer the factors that significantly influence the protein adsorption for the pure and the DOPC-covered interfaces.

**Table 2:** Properties of the Proteins

	lysozyme	cytochrome *c*	BSA
molecular weight (kDa)	14.3	12.4	66.5
molecular size (nm^3^)	3 × 3 × 4.5 ^25^	2.5 × 2.5 × 3.7 ^26^	4 × 4 × 14 ^27^
isoelectric point	11 ^28^	10 ^29^	5 ^30^
net charge at pH 7 ^31^	+7	+6	–18
surface hydrophobicity^31^	7.49		10.33
total hydrophobicity^31^	970	1110	1120
instability index	16.9 ^32^	15.56^a^	40.11 ^32^

a Calculated by ProtParam software (https://web.expasy.org/protparam). Proteins with instability indexes of <40 are predicted to be stable.

As described above, the *A*_a_ values for BSA and the other proteins were different, probably because of the different protein–tricaprylin and protein–DOPC interactions. However, the *A*_d_ value was independent of the type of protein (Table 1). Interestingly, the *A*_d_ values were very close to the area per lipid value previously reported for fully hydrated DOPC bilayers (67.4 Å^2^ at 30 °C).^24^ This suggests that the three proteins lost surface activity when the area per DOPC molecule decreased to the value for fully hydrated bilayers.

## Conclusions

We have reported a quantitative method to measure the dependence of protein adsorption and desorption at the tricaprylin/buffer interface on the area per DOPC molecule. We showed that the area per DOPC molecule required to prevent BSA adsorption was much smaller than that required to prevent lysozyme or cytochrome c adsorption. However, the area per DOPC molecule values required to remove all of the adsorbed protein molecules from the interface were almost the same, and these values were in good agreement with the area per lipid value reported for fully hydrated DOPC bilayers.

## Supplementary Material



## Notes

Surface pressure measurements of DOPC monolayers with high tricaprylin concentrations at the air/buffer interface, experimental setup for pendant drop tensiometry, and summary of the symbols used for the measured values (PDF)
