# Supplementary material for: Quantifying the Thresholds of the Phospholipid Surface Density for Nonspecific Protein Adsorption and Desorption at the Triacylglycerol/Water Interface
Source: Langmuir. 2025 Sep 12;41(37):25431–8. doi: 10.1021/acs.langmuir.5c03216 (PMC12461928; doi:10.1021/acs.langmuir.5c03216)
Supplement: Supplementary file 1 [file la5c03216_si_001.pdf]

Quantifying the Thresholds of the Phospholipid Surface Density for Nonspecific Protein Adsorption and Desorption at the Triacylglycerol/Water Interface

Chiho Kataoka-Hamai*

Research Center for Macromolecules and Biomaterials, National Institute for Materials Science, 1-1 Namiki, Tsukuba, Ibaraki 305-0044, Japan

*E-mail: kataoka.chiho@nims.go.jp

SUPPORTING INFORMATION

Surface Pressure Measurements of DOPC Monolayers with High Tricaprylin Concentrations at the Air/Buffer Interface. DOPC monolayers containing 20%–40% tricaprylin were compressed at a rate of 15 mm/min (Experimental Section). The resultant surface pressure curves (Figure S1, black) exhibited a shoulder at which the slope of the curve suddenly changed. Further compression after these shoulders resulted in the data overlapping. This behavior was consistent with previous results of egg PC/triolein monolayers and 1-palmitoyl-2-oleoyl-sn-glycero-3-phosphocholine/tricaprylin monolayers at the air/water interface.[1,2] Thus, the present data seem to be valid. However, for DOPC monolayers containing 50% tricaprylin, a compression rate of 15 mm/min appeared to be too slow to keep the monolayers stable. At this compression rate, the surface pressure after the shoulder was lower than those recorded for 20%–40% tricaprylin (Figure S1, green). This indicated that some of the DOPC molecules were removed from the monolayer during the compression. To avoid this lipid loss, we increased the compression rate from 15 to 45 mm/min (Figure S1, orange, blue, and red). As the rate increased, the surface pressure recovered and eventually overlapped with the data for 20%–40% tricaprylin. We therefore chose a compression rate of 45 mm/min for the DOPC monolayers with 50% tricaprylin. We also performed similar experiments for DOPC monolayers with 60% tricaprylin. For these monolayers, we chose a compression rate of 75 mm/min.

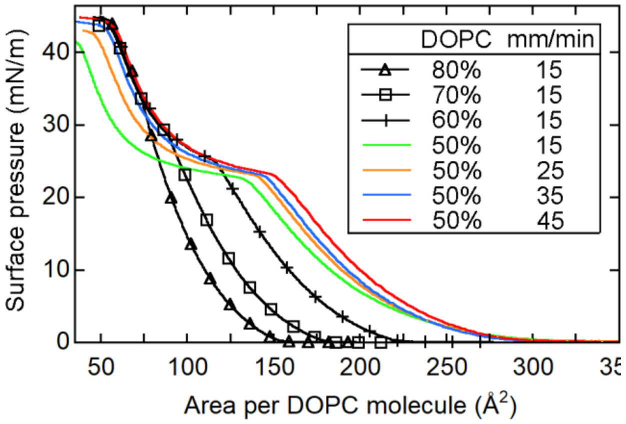

Figure S1. Dependence of the surface pressure on the compression rate for DOPC/tricaprylin monolayers at the air/buffer interface. The DOPC concentrations and the compression rates are shown in the graph.

Experimental Setup for the Pendant Drop Tensiometry. When the continuous phase in the measurement cell was replaced with pure buffer, fresh buffer was flowed into the cell using a peristaltic pump (Figure S2, red). The excess buffer was removed from the cell through an outlet needle connected to a suction pump (orange). The position of the end of this needle was fixed such that the solution volume in the cell was kept constant during each measurement. For the protein adsorption on the DOPC-covered interfaces, a protein solution was added from an inlet (green) and the solution was mixed with a magnetic stirrer.

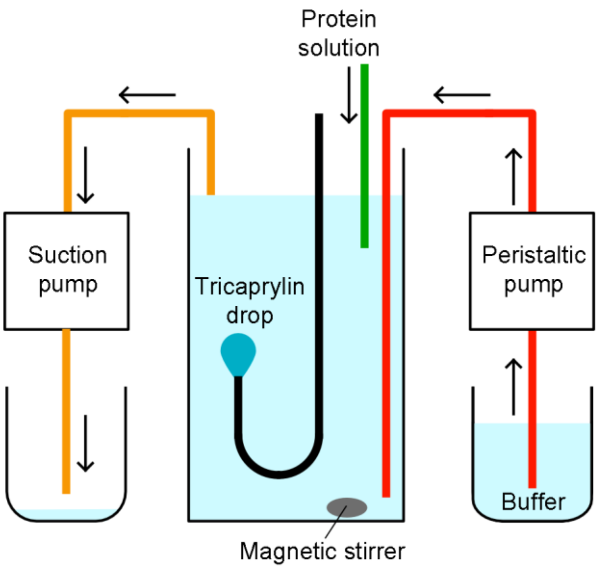

Figure S2. Experimental setup for the pendant drop tensiometry experiments. The continuous phase was replaced with pure buffer by flowing it into the measurement cell through a peristaltic pump. The excess buffer was removed with a suction pump.

Measured Values. The measured values and their symbols are summarized in Table S1.

Table S1. Measured values and their symbols.

■ Values determined in this study

Symbol	Definition
A_a	Threshold area per DOPC molecule that prevents protein adsorption (Figure 1, Table 1)
A_d	Threshold area per DOPC molecule at which adsorbed protein molecules completely desorb (Figure 1, Table 1)

■ Values used for determining the dependence of the interfacial tension at the tricaprylin/buffer interface on the area per DOPC molecule

Symbol	Definition
Π	Surface pressure of DOPC/tricaprylin monolayers at the air/buffer interface in the region in which the data in Figure 2A overlapped (Eq. 1)
Π_0	Collapse pressure of the pure tricaprylin monolayer at the air/buffer interface (Eq. 1)
γ	Interfacial tension at the tricaprylin/buffer interface that varies with the area per DOPC molecule (Eq. 1, Figure 2C)
γ_0	Interfacial tension at the pure tricaprylin/buffer interface (Eq. 1)

■ Values used for investigating protein adsorption at the pure tricaprylin/buffer interface

Symbol	Definition
$\Delta\gamma_{pads}$	Interfacial tension change caused by the adsorption of protein (2 μM) for 30 min at the pure tricaprylin/buffer interface (Figure 3, Table 1)

■ Values used for determining A_a

Symbol	Definition
γ_i	Interfacial tension after vesicle adsorption (0.07 mM lipid) and the successive removal of excess vesicles (Figure 4A)
$\Delta\gamma$	Interfacial tension change caused by the adsorption of protein (2 μM) for 30 min at the DOPC-coated interface in the absence of free vesicles (Figure 4A)
γ_i'	Interfacial tension after vesicle adsorption (0.07 mM lipid) and the successive drop volume decrease (Figure 4B). After the measurement of γ_i', protein was added without removing excess vesicles
$\Delta\gamma'$	Interfacial tension change caused by the adsorption of protein (2 μM) for 30 min at the DOPC-coated interface in the presence of free vesicles (Figure 4B, Eq. 2)
$\Delta\gamma_b$	Interfacial tension change in the absence of protein caused by DOPC adsorption (0.07 mM lipid) at the DOPC-coated interface with an interfacial tension of γ_i' (Figure 4C). $\Delta\gamma_b$ was used to calculate $\Delta\gamma$ from $\Delta\gamma'$ (Eq. 2)
$\gamma_{i,0}$	Interfacial tension at an area per DOPC molecule of A_a (Figure 4D)

■ Values used for determining A_d

Symbol	Definition
γ_{bp}	Interfacial tension at the DOPC-coated interface immediately before protein addition (Figures 5A and 7A)

References

(1) Kataoka-Hamai, C.; Kawakami, K. Determining the Dependence of Interfacial Tension on Molecular Area for Phospholipid Monolayers Formed at Silicone Oil–Water and Tricaprylin–Water Interfaces by Vesicle Fusion. *Langmuir* **2021**, *37*, 7527–7535.

(2) Mitsche, M. A.; Wang, L. B.; Small, D. M. Adsorption of Egg Phosphatidylcholine to an Air/Water and Triolein/Water Bubble Interface: Use of the 2-Dimensional Phase Rule To Estimate the Surface Composition of a Phospholipid/Triolein/Water Surface as a Function of Surface Pressure. *J. Phys. Chem. B* **2010**, *114*, 3276–3284.
